# Comparison Between Catheter-Directed Sclerotherapy and Surgical Removal of Large Ovarian Endometriomas: A Retrospective, Single-Center Observational Study

**DOI:** 10.3390/jcm15051959

**Published:** 2026-03-04

**Authors:** Yun Soo Chung, Hae-Rim Kim, Jin Kyung Baek, Heeyon Kim, Bo Hyon Yun, Man-Deuk Kim, Yong Jae Lee, Seok Kyo Seo

**Affiliations:** 1 Department of Obstetrics and Gynecology, Severance Hospital, Yonsei University College of Medicine, Seoul 03722, Republic of Korea; espera88@yuhs.ac (Y.S.C.); nupy90@yuhs.ac (J.K.B.); kimhy@yuhs.ac (H.K.); garfieldzz@yuhs.ac (B.H.Y.); 2 Institute of Women’s Life Medical Science, Yonsei University College of Medicine, Seoul 03722, Republic of Korea; 3 Department of Statistics, University of Seoul, Seoul 02504, Republic of Korea; haley2203@naver.com; 4 Department of Radiology, Severance Hospital, Yonsei University College of Medicine, Seoul 03722, Republic of Korea; mdkim@yuhs.ac; 5 Institute for Innovation in Digital Healthcare, Yonsei University, Seoul 03722, Republic of Korea

**Keywords:** cystectomy, endometrioma, sclerotherapy, oophorectomy, ovarian reserve

## Abstract

**Background/Objectives**: The treatment options for endometriosis vary depending on individual needs and clinical circumstances. To preserve ovarian reserve, ethanol catheter-directed sclerotherapy may be considered as a treatment option. We compared the efficacy of catheter-directed sclerotherapy with that of surgical removal for the treatment of large ovarian endometriomas. **Methods**: This retrospective, single-center study was conducted at a tertiary care center. Patients diagnosed with ovarian endometriomas of >10 cm between 1 January 2019 and 5 December 2024 were included. Fifteen patients underwent catheter-directed sclerotherapy, and 69 underwent laparoscopic ovarian cystectomy or oophorectomy. The changes in ovarian cyst size, anti-Müllerian hormone levels, and cancer antigen 125 levels after six months of treatment were determined. **Results**: Before matched comparison, anti-Müllerian hormone levels decreased from 2.48 ng/mL to 1.11 ng/mL 6 months after surgical treatment. In the catheter-directed sclerotherapy group, anti-Müllerian hormone levels decreased from 1.33 ng/mL to 1.19 ng/mL. In the 1:1 matched comparison between the catheter-directed sclerotherapy and surgical groups, the anti-Müllerian hormone levels decreased by approximately −0.13 and −0.59 in the catheter-directed sclerotherapy and surgical groups, respectively. The relative reduction in the anti-Müllerian hormone levels was approximately −0.25 and −0.78 in the unilateral and bilateral ovarian surgery groups, respectively. In the surgical group, cyst size decreased to 0 cm six months after treatment, whereas CA-125 levels decreased from 62.10 U/mL to 11.20 U/mL. In the CDS group, cyst size reduced to 3.30 cm, whereas CA-125 levels decreased from 74.20 U/mL to 17.60 U/mL. **Conclusions**: Catheter-directed sclerotherapy preserves ovarian reserve more effectively than surgical treatment, even in cases of large endometriomas. It may be a promising treatment option for individuals with low anti-Müllerian hormone levels who are planning to conceive.

## 1. Introduction

Endometriosis is the presence of endometrial glands and stroma outside the uterine cavity, particularly in the ovaries, rectovaginal septum, and pelvic peritoneum [1,2,3]. Approximately 6–10% of women in their reproductive ages have endometriosis, and up to 50% of women with infertility experience this condition [3,4]. Between 17% and 44% of patients with endometriosis develop endometriomas [5]—cystic lesions lined with endometrial mucosa resulting from recurrent hemorrhages [6]. Endometriomas are associated with a 0.3–0.8% risk of malignancy [7]. Endometriosis diagnosis is confirmed through vaginal examination and imaging techniques, such as ultrasonography and magnetic resonance imaging [8]. The treatment options for endometriosis vary depending on individual needs and circumstances. Nonsteroidal anti-inflammatory drugs, analgesics, hormonal treatments, and surgery are the most common options [8,9]. Surgery and medically assisted reproduction, rather than hormonal treatments, are recommended for endometriosis-related infertility [8]. For patients with symptoms related to endometriosis or those with large or rapidly enlarging endometriomas, concerns regarding malignancy, or complications with ovarian follicular access during in vitro fertilization (IVF), laparoscopic stripping may be recommended, despite the potential risk of a reduced ovarian reserve [10,11,12].

The European Society of Human Reproduction and Embryology guidelines suggest laparoscopic ovarian cystectomy for endometriomas of >4 cm for histological confirmation and to improve the ovarian response to IVF [13]. Medical management with close imaging and monitoring is recommended for smaller endometriomas (<3–4 cm) [12]. Surgical treatment may involve hemostasis using energy, sutures, or hemostatic agents; however, these methods are associated with increased ovarian damage. Cyst drainage and ethanol catheter-directed sclerotherapy (CDS) are considered less invasive alternative approaches for the removal of endometriomas [12]. However, the recurrence rates after cyst drainage are 80–100% [14]. Ethanol CDS preserves the ovarian reserve by targeting endometriomas without harming normal ovarian tissue [6]. Some studies have suggested that ethanol CDS reduces complications after surgical treatment, and its recurrence rate is comparable to that of conventional surgery [15].

Most endometriomas are <6 cm in diameter; however, ovarian endometriomas >10 cm have been observed, which are considered giant endometriomas [16,17]. Owing to their size, ovarian cysts >10 cm should raise suspicion for malignancy [18,19]. The rarity of giant endometriomas complicates their diagnosis and treatment [16]. Given that the treatment outcomes for this condition have not been extensively studied, in this study, we aimed to compare the effects of CDS and surgical treatment for giant endometriomas.

## 2. Materials and Methods

### 2.1. Study Participants and Selection Criteria

This was a retrospective, single-institution analysis conducted at Severance Hospital, Seoul, Republic of Korea, between 1 January 2019 and 5 December 2024. Since menopausal transition begins at an average age of 46 years [20], the inclusion criteria for this study were ages 18–45 years to ensure the inclusion of reproductive-aged women and those considering pregnancy and endometriomas >10 cm.

Owing to the retrospective nature of this study, the selection between CDS and surgery was not based on strict guidelines. However, after reviewing the participants’ medical records, we found that even in cases of large endometriomas, the main reasons for choosing CDS were patients’ preference for conservative treatment over surgery, low AMH levels due to a history of surgical treatment, recommendation of CDS by a local clinic, and referral to a radiologist before consultation with a gynecologist.

The exclusion criteria included a diagnosis of malignancy, polycystic ovarian syndrome, and missing baseline data on anti-Müllerian hormone (AMH) levels, ovarian cyst size, or cancer antigen 125 (CA-125) levels. To avoid potential confounding by patients with very low ovarian reserve, those with AMH levels below 0.7 ng/mL were excluded. Although AMH levels have not been definitively established as predictors of pregnancy, previous research has revealed that low AMH levels (0.2–0.7 ng/mL) demonstrate 40–97% sensitivity and 78–92% specificity for predicting response to stimulation in the general IVF population [20]. Although we did not aim to evaluate the IVF response in this study, we excluded women with AMH levels <0.7 ng/mL to focus on the treatment options for fertile women. By excluding patients with AMH levels <0.7 ng/mL, the potential confounding effect of severely diminished ovarian reserve on treatment outcomes was eliminated.

**Figure 1:**
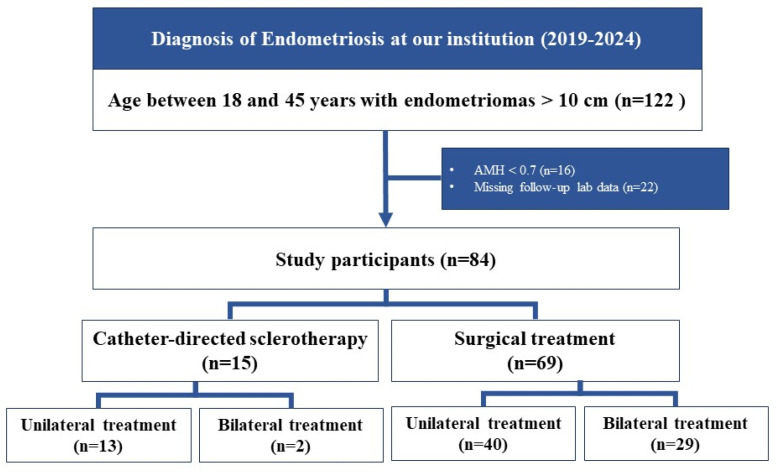
Flowchart of study participant selection.

Eighty-four participants had endometriomas of >10 cm, among whom 69 underwent surgical treatment and 15 received CDS. Among the 84 participants, 29 received surgical treatment for both ovaries, 40 received surgical treatment for a unilateral ovary, 13 received unilateral CDS treatment, and 2 received CDS treatment for both ovaries (Figure 1).

### 2.2. Variables

In this retrospective study, the following data were obtained from the participants: age, endometrioma laterality (unilateral or bilateral), body mass index (BMI), diabetes mellitus, alcohol consumption, smoking status, endometrioma size, AMH and CA-125 levels, and history of endometriosis recurrence. AMH was considered because it is currently the most reliable indicator of ovarian reserve in reproductive-aged women [21]. CA-125 was analyzed because it is a biomarker of endometriosis [22]. AMH and CA-125 levels and endometrioma size were assessed 6 months after treatment. Changes were calculated as the differences between the values after and before treatment, divided by the initial values. Relative percentage changes were reported rather than raw ratios.

$$Change\;in\;variables\;=\;\frac{Value\;after\;6\;months\;-\;Initial\;value\;before\;treatment}{Initial\;value\;before\;treatment}$$

For the surgical group, operation duration, estimated blood loss, changes in hemoglobin (Hb) levels preoperatively and on postoperative day one, and hospitalization length were analyzed. For the CDS group, procedure duration, hospitalization length, and estimated blood loss were analyzed.

Endometriomas were categorized as bilateral or unilateral. The size of the endometriomas was recorded in centimeters (cm) by measuring the longest length of the right or left ovarian cyst. The same ovaries were measured before treatment initiation and 6 months after treatment. Serum AMH and CA-125 levels were measured. Operational notes were used to collect data regarding operation duration and estimated blood loss. Hb changes were calculated by subtracting postoperative day one values from preoperative values.

### 2.3. Procedure

Experienced interventional radiologists performed the CDS. The endometriomas were initially identified using sonography or magnetic resonance imaging. Depending on the location and accessibility of the ovarian cysts, the intervention was performed transabdominally or transvaginally. During the procedure, the endometrioma was visualized using sonography. The endometrioma was punctured using an 18-gauge, 20 cm needle (Chiba Biopsy Needle; Cook, Bloomington, IN, USA) or an 18-gauge, 15 cm needle (Chiba Biopsy Needle; M.I. Tech, Gyeonggi-do, Republic of Korea). For the transvaginal approach, patients were placed in the lithotomy position, and the vagina was sterilized with povidone–iodine. Using transvaginal ultrasound guidance (LOGIQ E9; General Electric, Waukesha, WI, USA), an in-plane needle guidance adaptor was attached to the probe, and the endometrioma was targeted. After puncturing the endometrioma, a 0.035 in hydrophilic guidewire (Terumo, Tokyo, Japan) was advanced into the lesion, and the needle was replaced with an 8F pigtail catheter (Dawson–Mueller Drainage Catheter; Cook, Bloomington, IN, USA). Chocolate-colored fluid was aspirated, and the cyst was filled with contrast media to assess spillage into the pelvic cavity. After aspirating the contrast medium, 95% ethanol, at a volume equal to 25% of the aspirated volume, was infused into the cyst.

The patients were instructed to change positions (supine, bilateral decubitus, and prone) every 5 min, for a total of 20 min, while the catheter was clamped. After each position change, ethanol was aspirated until the injected volume was removed, and the catheter was removed. All patients were admitted one day before the procedure and discharged the day after CDS. Therefore, each case involved a total hospital stay of three days.

The aspirated chocolate-colored fluid was sent to the pathology department for cytological examination for malignancy. For the transabdominal approach, patients were placed in the supine position, and the area was sterilized with povidone–iodine. The remaining steps were the same as those in the transvaginal approach.

Obstetrics and gynecology experts performed the laparoscopic surgery. Laparoscopic oophorectomy was performed when malignancy was suspected before surgery, based on the CA-125 levels, endometrioma morphology, or intraoperative findings, such as a frozen section suggesting malignancy or severe adhesions, which ruled out laparoscopic cystectomy. In the other cases, laparoscopic cystectomy was performed. The endometriomas were identified using a laparoscopic camera after insufflating the abdominal cavity with CO_2_ gas. An incision was made into the endometrioma to remove the cysts from the ovary. A laparoscopic bag was used to extract the ovarian cyst through the umbilicus. The operation was completed after bleeding was controlled. For bleeding control, 16 cases were managed by suturing the ovarian tissue, whereas in the remaining cases, hemostasis was achieved using diathermy coagulation, and hemostatic agents were applied inside the ovary. The ovarian cysts were sent to the pathology department for endometriosis diagnosis.

### 2.4. Statistical Analysis

For continuous variables such as age, BMI, cyst size, AMH and CA-125 levels, operation duration, estimated blood loss, changes in Hb levels, and hospitalization length, data are represented as medians (Q1, Q3) for comparison. Since the data did not follow a normal distribution, as assessed by the Shapiro–Wilk test, the Wilcoxon test was performed. For categorical variables, such as diabetes mellitus, alcohol consumption, smoking status, and history of endometriosis, data are represented as numbers (%), and the chi-square test was used for analyses.

To compare the effects of CDS and surgery on ovarian reserve preservation while minimizing the confounding variables, a matched-pair test was performed under propensity score matching (PSM) using age, AMH levels before treatment, and type of ovarian cyst (unilateral or bilateral) as matched variables. AMH levels and age were considered because they are closely related to ovarian reserve and fertility [23]. Unilateral or bilateral ovarian cysts were analyzed because, according to previous research, operating on both ovaries affects the reduction in AMH levels more than unilateral ovary operations [24]. When comparing the effects of CDS and surgery, 1:1 and 1:2 matchings were conducted. For matching, the nearest-neighbor method with a standard mean difference of ≤0.05 was used. However, owing to the limited number of procedures involving right and left ovarian cysts, only 1:1 matching was performed when comparing CDS to surgery in the unilateral or bilateral ovaries.

Statistical significance was set at *p* < 0.05. All statistical analyses were performed using R software version 4.3.1 (The R Foundation, www.R-project.org, Vienna, Austria).

## 3. Results

### 3.1. Study Population

Age, BMI, diabetes mellitus, alcohol consumption, smoking status, endometrioma laterality (unilateral or bilateral), history of endometriosis recurrence, endometrioma size, and initial AMH and CA-125 levels were compared, and no significant differences were observed between the CDS and surgical groups (*p* > 0.050; Table 1). To compare the effects of CDS and surgical treatment in patients with similar ovarian reservoir statuses, PSM was conducted based on age, AMH levels, and type of ovarian cyst (unilateral or bilateral). Even after 1:1 and 1:2 paired matchings, the basic characteristics of the CDS and surgical groups showed no significant differences (*p* > 0.050; Supplementary Table S1). Because all participants underwent treatment for endometriomas, and previous studies have reported that endometriomas are a form of deep infiltrating endometriosis typically associated with at least stage III disease [25], most participants were assumed to have stage III or higher endometriosis based on their clinical histories.

To compare the effects of the unilateral and bilateral treatments in the surgical group, a 1:1 paired matching was conducted after PSM based on age and AMH levels. No significant differences were observed (*p* > 0.050; Supplementary Table S2).

### 3.2. CDS and Surgical Group

**Figure 2:**
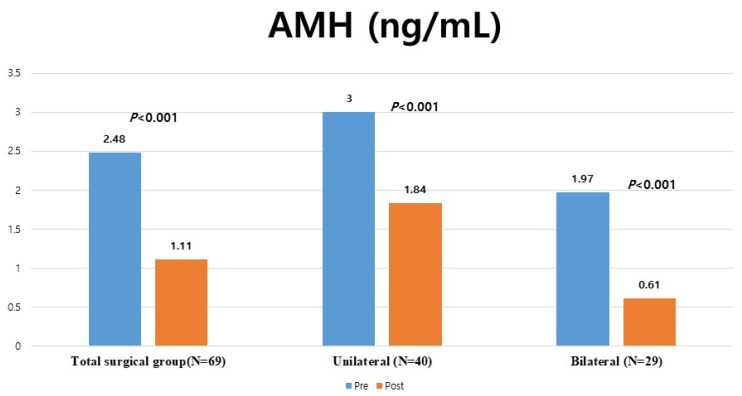
AMH levels before and after surgical treatments. The Y-axis represents the AMH levels (ng/mL), and the X-axis displays the different treatment groups. *p*-values and median AMH levels are reported above each graph. Blue bars represent the pre-treatment levels, and orange bars represent the post-treatment levels. AMH, anti-Müllerian hormone; N, number of participants in each treatment group. As shown in Table 3, when comparing the reduction in the relative ratio, before PSM, the AMH levels decreased by approximately −0.13 in the CDS group and −0.53 in the surgical group (*p* = 0.028). The CA-125 levels decreased by −0.79 in the CDS group and −0.85 in the surgical group (*p* = 0.312). We performed 1:1 and 1:2 PSM based on age, baseline AMH levels, and type of ovarian cyst (unilateral or bilateral). After 6 months of treatment, changes in ovarian cyst size, AMH levels, and CA-125 levels were compared. In the 1:1 matched comparison, the median reductions in ovarian cyst size were −0.71 and −1.00 in the CDS and surgical groups, respectively (*p* = 0.023). The AMH levels decreased by −0.13 in the CDS group and −0.59 in the surgical group (*p* = 0.006). The CA-125 levels exhibited no significant differences between the CDS and surgical groups (*p* = 0.232). Similar trends were observed in the 1:2 matched comparisons. Significant differences were observed in the changes in AMH levels and cyst size between the CDS and surgical groups. The AMH levels decreased by −0.13 and −0.59 in the CDS and surgical groups, respectively (*p* < 0.001). The changes in CA-125 levels showed no significant differences (*p* = 0.202).

As shown in Table 2, in the surgical group, the size of the endometriomas decreased from 10.50 cm before treatment to 0.00 cm 6 months after treatment (*p* < 0.001). The AMH levels decreased from 2.48 ng/mL to 1.11 ng/mL (*p* < 0.001, Figure 2). The CA-125 levels decreased from 62.10 U/mL to 11.20 U/mL (*p* < 0.001).

In the CDS group, the cyst size reduced from 10.00 cm to 3.30 cm 6 months after treatment (*p* < 0.001; Table 2). The AMH levels decreased from 1.33 ng/mL to 1.19 ng/mL, showing no significant difference (*p* = 0.322; Table 2). The CA-125 levels decreased from 74.20 U/mL to 17.60 U/mL (*p* = 0.001; Table 2).

The median operation time was 2 h and 23 min, whereas the median procedure time for CDS was 35 min. After CDS, patients were instructed to change their position for a total of 20 min following the procedure. The estimated blood loss was 100 cc for surgical cases, whereas it was minimal for CDS. No complications related to hemorrhage or infection were reported following either surgical or CDS treatments.

**Table 3:** Changes in AMH, CA-125, and cyst size between surgical and CDS groups with propensity-matched comparison.

Variable	Before PSM			1:1 Matching			1:2 Matching		
	CDS (N = 15)	Surgery (N = 69)	*p*-Value	CDS (N = 10)	Surgery (N = 10)	*p*-Value	CDS (N = 10)	Surgery (N = 20)	*p*-Value
Size of cysts (cm)	−0.74 (−0.89, −0.63)	−1.00 (−1.00, −1.00)	<0.001	−0.71 (−1.00, −0.63)	−1.00 (−1.00, −1.00)	0.023	−0.71 (−1.00, −0.63)	−1.00 (−1.00, −1.00)	0.001
AMH (ng/mL)	−0.13 (−0.52, 0.06)	−0.53 (−0.76, −0.28)	0.028	−0.13 (−0.52, 0.06)	−0.59 (−0.86, 0.00)	0.006	−0.13 (−0.52, 0.06)	−0.59 (−0.81, −0.25)	<0.001
CA-125 (U/mL)	−0.79 (−0.89, −0.52)	−0.85 (−0.94, −0.73)	0.312	−0.79 (−0.88, −0.57)	−0.89 (−0.95, −0.81)	0.232	−0.79 (−0.88, −0.57)	−0.85 (−0.93, −0.76)	0.202

Continuous variables are presented as median (Q1, Q3). The Wilcoxon test was performed. Data presented in bold indicates statistical significance. A paired Wilcoxon test was performed following a 1:1 matched control study of propensity score matching, using age, AMH levels, and the type of ovarian cyst (unilateral or bilateral). AMH, anti-Müllerian hormone; CA-125, cancer antigen 125; CDS, catheter-directed sclerotherapy; N, number.

$$Change\;in\;variables\;=\;\frac{Value\;after\;6\;months\;-\;Initial\;value\;before\;treatment}{Initial\;value\;before\;treatment}$$

### 3.3. Bilateral vs. Unilateral Interventions

In the surgical group, 40 patients were treated for unilateral ovarian cysts, and 29 were treated for bilateral ovarian cysts. The AMH levels of the unilateral surgical group decreased from 3.00 ng/mL to 1.84 ng/mL (*p* < 0.001; Table 2 and Figure 2). In the bilateral surgical group, the AMH levels were 1.97 ng/mL before treatment and 0.61 ng/mL 6 months after treatment (*p* < 0.001; Table 2 and Figure 2).

Among the 15 patients treated using CDS, 13 received treatment for unilateral ovaries and two for bilateral ovaries. Owing to the small sample size, comparisons between the unilateral and bilateral cases in CDS were not meaningful. The cases involving unilateral ovarian CDS only are included in Supplementary Table S3.

To assess the effects of unilateral vs. bilateral treatment on changes in ovarian cyst size, AMH levels, and CA-125 levels, a 1:1 PSM based on age and baseline AMH levels was performed. No significant differences in ovarian cyst size were observed between the two groups (*p* = 0.423; Table 4). However, the median relative reduction ratio in the AMH levels was −0.25 in the unilateral group and −0.78 in the bilateral ovarian cyst-treated group (*p* = 0.020; Table 4). The median relative reduction ratio of the CA-125 levels was −0.85 in the unilateral and bilateral ovarian cyst-treated groups (*p* = 0.846, Table 4). To determine whether changes in the AMH levels were influenced by the surgical outcomes, the operation duration, estimated blood loss, changes in Hb levels, and hospitalization length were compared. No significant differences were observed between the unilateral and bilateral surgical groups (*p* > 0.050; Supplementary Table S4).

$$Change\;in\;variables\;=\;\frac{Value\;after\;6\;months\;-\;Initial\;value\;before\;treatment}{Initial\;value\;before\;treatment}$$

## 4. Discussion

The results of this study showed that surgical treatment significantly decreased serum AMH levels 6 months postoperatively. By contrast, no significant reduction in AMH levels was observed in the CDS treatment group. AMH is strongly associated with the ovarian reserve, and the loss of healthy ovarian tissue and iatrogenic damage to the ovarian cortex may contribute to this decline after surgery [12]. As CDS does not harm the normal ovarian tissue, it is a promising alternative to surgery for preserving ovarian reserve and reducing surgical complications, particularly in women of reproductive age [6,12,15,26]. In women presenting with pelvic pain and subfertility, CA-125 levels higher than 30 U/mL are closely associated with endometriosis [22]. Although low CA-125 levels cannot definitively rule out endometriosis [22], CA-125 levels are considered a reliable marker, which significantly reduces after surgery [27]. Consistent with these findings, our study demonstrated a post-treatment decline in CA-125 levels to <30 U/mL. Moreover, previous studies have indicated that bilateral ovarian surgery increases the risk of premature ovarian failure [28]. Consistent with these findings, our study showed that patients undergoing bilateral surgical treatment experienced a greater decline in AMH levels even when age, surgical conditions, and baseline ovarian reserve were similar.

The treatment choice for endometriomas depends on patient factors such as age, family planning, and associated symptoms, including pelvic pain, dysmenorrhea, and dyspareunia [29]. Most endometriomas are <6 cm in diameter [16,17]. The malignant transformation of endometriomas is relatively rare (approximately 0.3–0.8%) [7]; surgical treatment is preferred when endometriomas exceed 10 cm in diameter because of their large size [16]. Although our findings suggest that CDS could be considered even in cases of endometriomas >10 cm, current guidelines still favor surgery. Some studies suggest that large endometriomas may result in poor reproductive outcomes following IVF. Therefore, surgical treatment is recommended when low-quality oocytes or embryos are repeatedly observed during IVF cycles [30]. However, other studies, including several meta-analyses, do not fully support the notion that surgical treatment before IVF improves reproductive outcomes [31]. As surgical treatment potentially decreases ovarian function [32] in women desiring pregnancy, the decision to perform surgery should involve assessing the potential benefit of fertility against the risk of damaging the ovarian reserve [28].

This study was limited by its small sample size and single-institution design. Although a large-scale randomized controlled trial would be valuable to strengthen these findings, the rarity of endometriomas >10 cm makes the findings of this study important. However, the small sample size and imbalance between the CDS and surgical cases could decrease the statistical power. To address this concern, we implemented PSM and adjustments; however, this is not a perfect solution. According to previous studies, recurrence rates following CDS are controversial. While several studies have reported that recurrence rates following ethanol-retained CDS are comparable to those following surgical treatment [33,34], others suggest that CDS carries a higher recurrence risk than surgical treatment [35,36]. The recurrence rates after CDS vary depending on the material used for sclerotherapy and the duration of instillation [34]. One study reported that for ethanol sclerotherapy with a 10 min instillation, the recurrence rate was 9.1%, whereas the recurrence rate for surgical treatment was 3.8% [36]. Since this study lacked long-term follow-up because most patients were referred back to the local clinics or lost to follow-up, further studies are needed to better evaluate the differences in recurrence rates, treatment satisfaction, and changes in pain scores between CDS and surgical treatments. Even under this condition, in the surgical group, 35 patients (50.72%) reported no prior sexual activity, were unmarried, or had no plans for pregnancy; 17 (24.64%) were lost to follow-up, mostly due to residence; 12 (17.39%) were referred out; 4 (5.80%) delivered babies; and 1 (1.45%) patient is currently trying to conceive. In the CDS group, 4 (26.67%) were lost to follow-up; 9 (60%) were still with no prior sexual activity, unmarried, or had no plans for pregnancy; and 2 (13.33%) had deliveries. In pregnant women, a previous study reported that spontaneous rupture of abnormal vascular proliferation could occur [37]. However, none of our pregnant cases in both the surgical and CDS groups had any complications during pregnancy. Another limitation of this study was the exclusion of patients with AMH levels <0.7 ng/mL. This may limit the generalizability to the broader infertile population; however, it may have reduced potential confounding by helping to distinguish the effects of diminished ovarian reserve from the effects of the treatment itself.

A major strength of this study was its focus on large endometriomas, an area rarely addressed in previous studies, especially aiding clinicians in managing sizable endometriomas in patients with fertility plans. Another strength is the use of matched-pair comparisons, which controlled for confounding factors that may influence ovarian reserve, supporting the validity of the findings.

## 5. Conclusions

Overall, CDS preserves ovarian reserve more effectively than surgical treatment, even in cases of large endometriomas. CDS may be a promising treatment option for individuals with low AMH levels who are planning to conceive. However, given the retrospective nature of this study and the small sample size, further long-term follow-up studies are required. Physicians should also carefully consider the potential benefits and risks of each treatment.

## Figures and Tables

**Table 1:** Basic participant characteristics between the CDS and surgical treatment groups.

Variable	CDS (N = 15)	Surgery (N = 69)	*p*-Value
Age (year)	31.00 (27.50, 35.00)	31.00 (27.00, 37.00)	0.958
BMI (kg/m^2^)	19.92 (18.54, 23.45)	21.15 (19.96, 23.95)	0.229
Diabetes mellitus	0 (0%)	0 (0%)	N/A
Alcohol	7 (46.67%)	28 (40.58%)	0.885
Smoking	1 (6.67%)	2 (2.9%)	1.000
Types of ovarian cysts			0.073
Unilateral	13 (86.67%)	40 (57.97%)	
Bilateral	2 (13.33%)	29 (42.03%)	
History of endometriosis recurrence	2 (13.33%)	4 (5.80%)	0.635
Size of cysts (cm)	10.00 (9.80, 11.50)	10.50 (10.00, 12.00)	0.242
AMH (ng/mL)	1.33 (0.98, 3.10)	2.48 (1.14, 4.31)	0.277
CA-125 (U/mL)	74.20 (53.60, 152.00)	62.10 (41.20, 111.00)	0.532

Continuous variables are presented as median (Q1, Q3), and categorical variables in N (%). The Wilcoxon test was performed for continuous variables, and the chi-square test was performed for categorical variables. AMH, anti-Müllerian hormone; BMI, body mass index; CA-125, cancer antigen 125; CDS, catheter-directed sclerotherapy; N, number; N/A, not applicable.

**Table 2:** Changes in AMH, CA-125, and cyst size in CDS vs. surgical treatment, including unilateral–bilateral comparisons.

	CDS Total			Surgery Total			Unilateral Ovary Surgery			Bilateral Ovaries Surgery		
	Pre (N = 15)	Post (N = 15)	*p*-Value	Pre (N = 69)	Post (N = 69)	*p*-Value	Pre (N = 40)	Post (N = 40)	*p*-Value	Pre (N = 29)	Post (N = 29)	*p*-Value
Size of cysts (cm)	10.00 (9.80, 11.50)	3.30 (1.20, 3.55)	**<0.001**	10.50 (10.00, 12.00)	0.00 (0.00, 0.00)	**<0.001**	10.75 (10.05, 12.15)	0.00 (0.00, 0.00)	**<0.001**	10.10 (9.80, 11.60)	0.00 (0.00, 0.00)	**<0.001**
AMH (ng/mL)	1.33 (0.98, 3.10)	1.19 (0.48, 2.86)	0.322	2.48 (1.14, 4.31)	1.11 (0.58, 2.37)	**<0.001**	3.00 (1.54, 4.66)	1.84 (0.90, 4.16)	**<0.001**	1.97 (0.81, 3.61)	0.61 (0.22, 1.03)	**<0.001**
CA-125 (U/mL)	74.20 (53.60, 152.0)	17.60 (14.20, 25.30)	**0.001**	62.10 (41.20, 111.00)	11.20 (8.20, 13.40)	**<0.001**	62.30 (40.75, 109.50)	10.40 (8.20, 13.40)	**<0.001**	62.00 (42.00, 155.00)	11.70 (8.65, 14.05)	0.063

Continuous variables are presented as median (Q1, Q3), and categorical variables in N (%). The Wilcoxon test was performed. Data presented in bold indicates statistical significance. AMH, anti-Müllerian hormone; CA-125, cancer antigen 125; CDS, catheter-directed sclerotherapy; Pre, pre-treatment; Post, 6 months after the treatment; N, number.

**Table 4:** Changes in AMH, CA-125, and cyst size between unilateral and bilateral surgical ovarian treatments post-matching.

	Unilateral (N = 10)	Bilateral (N = 10)	*p*-Value
Changes in size of cysts (cm)	−1.00 (−1.00, −100)	−1.00 (−1.00, −100)	0.423
Changes of AMH level (ng/mL)	−0.25 (−0.55, 0.06)	−0.78 (−0.87, −0.68)	**0.020**
Changes of CA-125 level (U/mL)	−0.85 (−0.94, −0.74)	−0.85 (−0.93, −0.80)	0.846

Continuous variables are presented as median (Q1, Q3). Data presented in bold indicates statistical significance. A paired Wilcoxon test was performed following a 1:1 matched control study of propensity score matching, using age and AMH levels. AMH, anti-Müllerian hormone; CA-125, cancer antigen 125; N, number.

## Data Availability

The dataset is available upon request from the authors.

## Supplementary Materials

The following supporting information can be downloaded at: https://www.mdpi.com/article/10.3390/jcm15051959/s1: Table S1. Basic participant characteristics between the catheter-directed sclerotherapy and surgical treatment groups after propensity score matching; Table S2. Basic characteristics of participants in the unilateral and bilateral ovarian cyst treatment groups of surgical treatment, before and after propensity score matching; Table S3. Changes in AMH and CA-125 levels, as well as cyst sizes, in the unilateral ovary CDS group; Table S4. Basic characteristics of participants in the unilateral and bilateral ovarian treatment groups of surgical treatment after propensity score matching.

## Abbreviations

The following abbreviations are used in this manuscript:

CDS	catheter-directed sclerotherapy
CA-125	cancer antigen 125
AMH	anti-Müllerian hormone
PSM	propensity score matching
IVF	in vitro fertilization
Hb	hemoglobin

## References

[1] Guidozzi F. (2021). Endometriosis-associated cancer. Climacteric.

[2] Pearce C.L., Templeman C., Rossing M.A., Lee A., Near A.M., Webb P.M., Nagle C.M., Doherty J.A., Cushing-Haugen K.L., Wicklund K.G. (2012). Association between endometriosis and risk of histological subtypes of ovarian cancer: A pooled analysis of case–control studies. Lancet Oncol..

[3] Giudice L.C. (2010). Clinical practice. Endometriosis. N. Engl. J. Med..

[4] Eskenazi B., Warner M.L. (1997). Epidemiology of endometriosis. Obstet. Gynecol. Clin. N. Am..

[5] Redwine D.B. (1999). Ovarian endometriosis: A marker for more extensive pelvic and intestinal disease. Fertil. Steril..

[6] Azizova A., Ciftci T.T., Gultekin M., Unal E., Akhan O., Bozdag G., Akinci D. (2024). Ethanol sclerotherapy in the management of ovarian endometrioma: Technical considerations for catheter- and needle-directed sclerotherapy. Cardiovasc. Intervent. Radiol..

[7] Yazbeck C., Koskas M., Scali S.C., Kahn V., Luton D., Madelenat P. (2012). How I do… ethanol sclerotherapy for ovarian endometriomas. Gynecol. Obstet. Fertil..

[8] Becker C.M., Bokor A., Heikinheimo O., Horne A., Jansen F., Kiesel L., King K., Kvaskoff M., Nap A., Petersen K. (2022). ESHRE guideline: Endometriosis. Hum. Reprod. Open.

[9] Park J., Chang H.J., Hwang K.J., Yum S.H., Park C.E., Kim J.H., Kim M. (2024). Association of COX-2 selectivity in pain medication use with endometriosis incidence: Retrospective cohort study. Yonsei Med. J..

[10] Cranney R., Condous G., Reid S. (2017). An update on the diagnosis, surgical management, and fertility outcomes for women with endometrioma. Acta Obstet. Gynecol. Scand..

[11] Deckers P., Ribeiro S.C., Simões R.S., Miyahara C.B.D.F., Baracat E.C. (2018). Systematic review and meta-analysis of the effect of bipolar electrocoagulation during laparoscopic ovarian endometrioma stripping on ovarian reserve. Int. J. Gynaecol. Obstet..

[12] Rehmer J.M., Flyckt R.L., Goodman L.R., Falcone T. (2019). Management of endometriomas. Obstet. Gynecol. Surv..

[13] Gelbaya T.A., Gordts S., D’Hooghe T.M., Gergolet M., Nardo L.G. (2010). Management of endometrioma prior to IVF: Compliance with ESHRE guidelines. Reprod. Biomed. Online.

[14] Donnez J., Nisolle M., Gillerot S., Anaf V., Clerckx-Braun F., Casanas-Roux F. (1994). Ovarian endometrial cysts: The role of gonadotropin-releasing hormone agonist and/or drainage. Fertil. Steril..

[15] Garcia-Tejedor A., Martinez-Garcia J.M., Candas B., Suarez E., Mañalich L., Gomez M., Merino E., Castellarnau M., Regueiro P., Carreras M. (2020). Ethanol sclerotherapy versus laparoscopic surgery for endometrioma treatment: A prospective, multicenter, cohort pilot study. J. Minim. Invasive Gynecol..

[16] Yahya A., Mustapha A., Kolawole A.O., Oguntayo A.O., Bello N., Aliyu H.O., Adewuyi S.A. (2021). Giant ovarian endometrioma: A case report. J. West Afr. Coll. Surg..

[17] Miranda J.A., Fabrini E., Coelho F.M.A., Viana P.C.C. (2024). Giant endometrioma in an asymptomatic patient. Radiol. Case Rep..

[18] Yaşar L., Süha Sönmez A., Galip Zebitay A., Neslihan G., Yazicioglu H.F., Mehmetoğlu G. (2010). Huge ovarian endometrioma—A case report. Gynecol. Surg..

[19] Reuter K., Davidoff A., Cooney J., Hunter R. (1988). An unusually large endometrioma simulating an ovarian malignancy. AJR Am. J. Roentgenol..

[20] Taylor H.S., Pal L., Seli E. (2020). Speroff’s Clinical Gynecologic Endocrinology and Infertility.

[21] Cedars M.I. (2022). Evaluation of female fertility—AMH and ovarian reserve testing. J. Clin. Endocrinol. Metab..

[22] Hirsch M., Duffy J.M., Deguara C.S., Davis C.J., Khan K.S. (2017). Diagnostic accuracy of cancer antigen 125 (CA125) for endometriosis in symptomatic women: A multicenter study. Eur. J. Obstet. Gynecol. Reprod. Biol..

[23] Shrikhande L., Shrikhande B., Shrikhande A. (2020). AMH and its clinical implications. J. Obstet. Gynecol. India.

[24] Younis J.S., Shapso N., Fleming R., Ben-Shlomo I., Izhaki I. (2018). Impact of unilateral versus bilateral ovarian endometriotic cystectomy on ovarian reserve: A systematic review and meta-analysis. Hum. Reprod. Update.

[25] Seraji S., Ali A., Demirel E., Akerman M., Nezhat C., Nezhat F.R. (2024). Association between ovarian endometriomas and stage of endometriosis. J. Clin. Med..

[26] Han K., Seo S.K., Kim M.-D., Kim G.M., Kwon J.H., Kim H.J., Won J.Y., Lee D.Y. (2018). Catheter-directed sclerotherapy for ovarian endometrioma: Short-term outcomes. Radiology.

[27] Sarbazi F., Akbari E., Karimi A., Nouri B., Noori Ardebili S.H. (2021). The clinical outcome of laparoscopic surgery for endometriosis on pain, ovarian reserve, and cancer antigen 125 (CA-125): A cohort study. Int. J. Fertil. Steril..

[28] Busacca M., Vignali M. (2009). Endometrioma excision and ovarian reserve: A dangerous relation. J. Minim. Invasive Gynecol..

[29] Muzii L., Di Tucci C., Di Feliciantonio M., Galati G., Verrelli L., Donato V.D., Marchetti C., Panici P.B. (2017). Management of endometriomas. Semin. Reprod. Med..

[30] Park H.J., Kim H., Lee G.H., Yoon T.K., Lee W.S. (2019). Could surgical management improve the IVF outcomes in infertile women with endometrioma? A review. Obstet. Gynecol. Sci..

[31] Ferrero S., Gazzo I., Crosa M., Rosato F.P., Barra F., Leone Roberti Maggiore U. (2024). Impact of surgery for endometriosis on the outcomes of in vitro fertilization. Best Pract. Res. Clin. Obstet. Gynaecol..

[32] Choi S.H., Kim S., Lee S.W., Won S., Shim S.H., Lee N., Kim M.K., Jung Y.W., Seong S.J., Kim M.L. (2023). Recurrence, reoperation, pregnancy rates, and risk factors for recurrence after ovarian endometrioma surgery: Long-term follow-up of 756 women. Yonsei Med. J..

[33] Jee B.C. (2022). Efficacy of ablation and sclerotherapy for the management of ovarian endometrioma: A narrative review. Clin. Exp. Reprod. Med..

[34] Cohen A., Almog B., Tulandi T. (2017). Sclerotherapy in the management of ovarian endometrioma: Systematic review and meta-analysis. Fertil. Steril..

[35] Dwi N. (2024). Sclerotherapy in the treatment of endometrioma: A comprehensive systematic review. Int. J. Med. Sci. Health Res..

[36] Noma J., Yoshida N. (2001). Efficacy of ethanol sclerotherapy for ovarian endometriomas. Int. J. Gynecol. Obstet..

[37] Vuong A.D.B., Pham T.H., Nguyen X.T., Trinh N.B., Nguyen P.N., Ho Q.N. (2023). Spontaneous hemoperitoneum in the second and third trimester of pregnancy: Two uncommon case reports at tu du hospital, in vietnam and a literature review. Int. J. Emerg. Med..

