# Supplementary material for: Comparison Between Catheter-Directed Sclerotherapy and Surgical Removal of Large Ovarian Endometriomas: A Retrospective, Single-Center Observational Study
Source: J Clin Med. 2026 Mar 4;15(5):1959. doi: 10.3390/jcm15051959 (PMC12986368; doi:10.3390/jcm15051959)
Supplement: Supplementary file 1 [file jcm-15-01959-s001.zip › jcm-4161317-supplementary.pdf]

Supplementary Table S1. Basic participant characteristics between catheter-directed sclerotherapy and surgical treatment group after propensity score matching.

Variable	1:1 matching			1:2 matching		
	CDS (N=10)	Surgery (N=10)	*P*-value	CDS (N=10)	Surgery (N=20)	*P*-value
Age (year)	31.00 (27.00, 34.00)	31.50 (27.00, 35.00)	0.849	31.00 (27.00, 34.00)	30.00 (28.00, 35.00)	0.982
BMI (kg/m^2)	20.53 (18.96, 23.12)	21.41 (20.20, 22.79)	0.436	20.53 (18.96, 23.12)	20.34 (19.24, 21.63)	1.00
Diabetes mellitus	0 (0%)	0 (0%)	N/A	0 (0%)	0 (0%)	N/A
Alcohol	5 (50.00%)	6 (60.00%)	1.000	5 (50.00%)	9 (45.00%)	1.000
Smoking	1 (10%)	0 (0%)	1.000	1 (10.00%)	0 (0.0%)	0.719
Types of ovarian cysts			1.000			0.236
Unilateral	8 (80.00%)	7 (70.00%)		8 (80.00%)	10 (50.00%)	
Bilateral	2 (20.00%)	3 (30.00%)		2 (20.00%)	10 (50.00%)	
History of endometriosis recurrence	0 (0.00%)	0 (0.00%)	1.000	0 (0.00%)	2 (10.00%)	0.796
Size of cysts (cm)	10.70 (9.90, 12.00)	10.40 (10.00, 11.60)	0.970	10.70 (9.90, 12.00)	10.30 (9.90, 11.60)	0.843
AMH (ng/mL)	1.24 (0.96, 2.67)	1.78 (0.69, 3.27)	0.796	1.24 (0.96, 2.67)	2.71 (1.42, 4.36)	0.169
CA-125 (U/mL)	78.60 (65.80, 162.00)	95.55 (70.90, 143.00)	0.739	78.60 (65.80, 162.00)	70.30 (49.40, 107.00)	0.502

Continuous variables are presented as median (Q1, Q3), and categorical variables in N (%). The Wilcoxon test was performed. Propensity score matching was conducted based on age, baseline AMH, and the type of ovarian cyst (unilateral or bilateral). *AMH* anti-Müllerian hormone; *CA-125* cancer antigen 125; *CDS* catheter-directed sclerotherapy; *N* number; *N/A* not applicable

Supplementary Table S2. Basic characteristics of participants in the unilateral and bilateral ovarian cyst treatment groups of surgical treatment, before and after propensity score matching

Variable	Before matching			After 1:1 matching		
	Unilateral (N=40)	Bilateral (N=29)	*P*-value	Unilateral (N=10)	Bilateral (N=10)	*P*-value
Age (year)	32.50 (27.00, 37.00)	30.00 (27.00, 35.00)	0.613	31.50 (27.00, 35.00)	30.00 (29.00, 35.00)	0.621
History of endometriosis recurrence	1 (2.50%)	3 (10.34%)	0.393	0 (0.00%)	2 (20.00%)	0.456
Size of cysts (cm)	10.75 (10.05, 12.15)	10.10 (9.80, 11.60)	0.109	10.60 (10.00, 12.00)	10.15 (9.80, 11.30)	0.363
AMH (ng/mL)	3.00 (1.54, 4.66)	1.97 (0.81, 3.61)	0.100	2.75 (1.25, 4.40)	2.71 (1.97, 3.27)	0.971
CA-125 (U/mL)	62.30 (40.75, 109.50)	62.00 (42.00, 155.0)	0.961	78.50 (53.00, 109.00)	67.65 (45.80, 80.80)	0.579

Continuous variables are presented as median (Q1, Q3), and categorical variables in N (%). The Wilcoxon test was performed. Propensity score matching was conducted based on age and baseline AMH. *AMH* anti-Müllerian hormone; *CA-125* cancer antigen 125; *N* number

Supplementary Table S3 Changes in AMH and CA-125 levels, as well as cyst sizes, in unilateral ovary CDS group.

Variable	Unilateral ovary CDS		
	Pre (N=13)	Post (N=13)	*P*-value
Size of cysts (cm)	10.00 (9.90,11.40)	2.60 (0.00, 3.40)	**0.016**
AMH (ng/mL)	1.33 (1.00, 3.53)	1.78 (0.62, 3.02)	0.641
CA-125 (U/mL)	74.20 (65.80, 142.00)	16.55 (12.80, 21.60)	**0.006**

Continuous variables are presented as median (Q1, Q3), and categorical variables in N (%). The Wilcoxon test was performed. Data presented in bold indicates statistical significance. *AMH* anti-Müllerian hormone; *CA-125* cancer antigen 125; *CDS* catheter-directed sclerotherapy; *Pre* pre-treatment; *Post* 6 months after the treatment; *N* number.

Supplementary Table S4. Basic characteristics of participants in the unilateral and bilateral ovarian treatment groups of surgical treatment after propensity score matching.

	Unilateral (N=10)	Bilateral (N=10)	*P*-value
Duration of operation (hours)	2.09 (1.65, 2.17)	2.86 (2.67, 4.08)	0.155
Estimated blood loss (cc)	50.00 (0.00, 100.00)	225 (150.00, 300.00)	0.153
Hb level (g/dL)	2.00 (1.00, 2.50)	2.50 (2.30, 2.70)	0.357
Duration of admission (days)	4.00 (3.00, 5.00)	4.50 (4.00, 5.00)	0.202

Continuous variables are presented as median (Q1, Q3). A paired Wilcoxon test was performed following 1:1 matched control study of propensity score matching, using age and AMH levels.
